# Attitudes of Anaesthesiology Specialists and Residents Toward Hemodynamic Monitoring: A National Survey Study

**DOI:** 10.4274/TJAR.2025.251940

**Published:** 2026-02-09

**Authors:** Gamze Talih, Aslıhan Aykut, Burhan Dost, Emre Sertaç Bingül, Başak Akça, Muhammed Enes Aydın, Z. Aslı Demir, Ümit Karadeniz, Ali Fuat Erdem

**Affiliations:** 1 Erciyes University Faculty of Medicine, Department of Anaesthesiology and Reanimation, Kayseri, Türkiye; 2 Ankara Bilkent City Hospital, Clinic of Anaesthesiology and Reanimation, Ankara, Türkiye; 3 Ondokuz Mayıs University Faculty of Medicine, Department of Anaesthesiology and Reanimation, Samsun, Türkiye; 4 İstanbul University, İstanbul Faculty of Medicine, Department of Anaesthesiology and Reanimation, İstanbul, Türkiye; 5 Hacettepe University Faculty of Medicine, Department of Anaesthesiology and Reanimation, Ankara, Türkiye; 6 Atatürk University Faculty of Medicine, Department of Anaesthesiology and Reanimation, Erzurum, Türkiye; 7 Sakarya University Faculty of Medicine, Department of Anaesthesiology and Reanimation, Sakarya, Türkiye

**Keywords:** Advanced hemodynamic monitoring, fluid responsiveness, goal-directed therapy, high-risk surgical patients, survey

## Abstract

**Objective:**

This descriptive survey study aims to evaluate the knowledge, attitudes, and practices of anaesthesiology specialists and residents in Türkiye regarding advanced hemodynamic monitoring in high-risk surgical patients.

**Methods:**

The survey, comprising 25 questions, was distributed to 960 anaesthesia professionals, with 713 completing the questionnaire.

**Results:**

The study reveals that while invasive blood pressure monitoring is widely used (96.3%), the adoption of advanced hemodynamic monitoring techniques, such as cardiac output monitoring, remains limited (12.6%). For awake high-risk surgical patients under regional anaesthesia, a significant proportion of respondents (15.1% and 37.1%) considered non-invasive blood pressure monitoring to be insufficient. Additionally, 41.1% of participants believed that stroke volume variation, pulse pressure variation, and systolic pressure variation parameters could be used to assess fluid deficits in awake patients.

**Conclusion:**

High costs, technical complexity, and lack of training are identified as major barriers. The findings highlight the need for enhanced educational programs and practical training to improve the utilization of advanced hemodynamic monitoring, ultimately aiming to reduce perioperative morbidity and mortality. The study underscores the importance of integrating advanced hemodynamic monitoring into routine clinical practice and suggests the development of nationwide algorithms to standardize practices.

**Main Points**

• The use of advanced hemodynamic monitoring techniques among anaesthesiologists in Türkiye is limited in high-risk surgical patients.

• High costs, technical complexity, and lack of experience are significant barriers to the widespread adoption of these techniques.

• Dynamic fluid responsiveness parameters and cardiac output monitoring preferences have fallen behind conventional monitoring methods in terms of usage rates in high-risk surgeries.

## Introduction

With advancements in medical technology and perioperative care, the number of patients undergoing high-risk or major surgeries has been increasing. Although high-risk surgeries constitute only 12.5% of all surgical procedures, they account for more than 80% of postoperative mortality.^1^ Hemodynamic instability is common during these procedures, making the optimization of hemodynamic management a crucial factor directly associated with patient outcomes. Effective hemodynamic monitoring helps detect physiological changes, diagnose underlying causes, and optimize oxygen delivery to tissues. Additionally, it is essential for assessing the adequacy of therapeutic interventions such as fluid resuscitation or vasoactive drug administration. Studies have shown that hemodynamic optimization in high-risk surgical patients reduces postoperative complications and improves overall outcomes.^2^

Beyond routine hemodynamic parameters such as blood pressure, urine output, and blood gas values, cardiac output (CO) monitoring and dynamic fluid responsiveness assessment can be utilized for intraoperative hemodynamic optimization. Various hemodynamic monitoring devices exist with different levels of invasiveness and accuracy. However, challenges such as high costs, technical complexity, and a lack of knowledge or experience hinder the widespread adoption of hemodynamic monitoring techniques. Surveys conducted among anaesthesiologists in North America, Europe, Korea, Italy, and China have revealed significant gaps in the clinical application of hemodynamic monitoring and management in high-risk surgeries.^3, 4, 5, 6^ Similarly, a study among anaesthesiologists in Japan indicated that while CO and dynamic fluid monitoring parameters were recommended for high-risk surgical patients, their actual use in clinical practice remained low.^7^

To date, no study has been conducted to investigate the frequency and details of hemodynamic monitoring among anaesthesiologists in Türkiye. This study aims to evaluate the monitoring methods used to ensure hemodynamic stability during and after surgery, analyze the knowledge, experience, and perspectives of anaesthesiologists regarding hemodynamic monitoring, determine the factors influencing device and technique preferences, assess critical thresholds considered in hemodynamic parameter monitoring, and identify potential educational or developmental needs in this field.

## Methods

This descriptive survey study was designed to evaluate the knowledge and attitudes of anaesthesiology specialists and residents in Türkiye regarding advanced hemodynamic monitoring in high-risk surgical patients. The study received approval from the Ankara Bilkent City Hospital, Scientific and Ethical Evaluation Board for Medical Research No. 2 (TABED) (approval no.: TABED 2-24-635, date: 13.11.2024). The survey was conducted using web-based software (SurveyMonkey, San Mateo, CA) and was available for participation between November 15-21, 2024. Informed consent was received from all participants.

The target population consisted of anaesthesiology specialists and residents working in Türkiye. According to the *Turkish Society of Anesthesiology and Reanimation*-TARD website, there are 4,438 anaesthesiology specialists and 5,110 residents. A web link to the survey (SurveyMonkey, Palo Alto, CA, USA) was sent to 960 participants (10% of the total population). Retired physicians were excluded from the study.

### Survey Structure

The survey comprised 25 questions divided into three main sections:

Descriptive and Socio-demographic Questions: This section collected basic demographic and professional data, including participants’ age, gender, years of professional experience, level of education, and workplace information.

Knowledge Assessment Questions: These questions aimed to evaluate participants’ knowledge of advanced hemodynamic monitoring practices in high-risk surgical patients.

Attitude Assessment Questions: This section assessed participants’ perspectives and attitudes toward advanced hemodynamic monitoring using statements rated on a five-point Likert scale (1: strongly disagree - 5: strongly agree) (Supplementary Form 1).

### Definition of High-Risk Surgical Patients

For this study, high-risk surgical patients were defined as adults (≥18 years) undergoing surgeries expected to last more than two hours and meeting at least two of the following criteria:

- Presence of functional limitation due to cardiac or respiratory disease.

- Extensive surgery planned for cancer requiring bowel anastomosis.

- Expected acute massive blood loss (>2 liters) during surgery.

- Age ≥65 years with functional impairment in one or more organ systems.

- Septicemia (positive blood cultures or septic focus).

- Open-heart surgery or complex cardiac procedures.

- Acute abdomen (e.g., pancreatitis, perforation, gastrointestinal bleeding).

- Acute kidney injury (creatinine >2 mg dL^-1^).

- Aortic surgery.

- Extensive malignancy surgery.

The criteria for patient and surgical classification were adapted from similar surveys conducted among North American and European anaesthesiologists.^3^ To ensure content appropriateness and clarity, the full questionnaire was reviewed by an expert.

### Statistical Analysis

Statistical analyses were performed using the Statistical Package for the Social Sciences software, version 23.0 (IBM Corp., Armonk, NY, USA). The collected data were analyzed using descriptive statistical methods. Categorical variables were presented as counts and percentage.

## Results

A total of 960 anaesthesia specialists and residents were invited to participate in the survey, of whom 713 completed the questionnaire. The demographic characteristics of the participants are presented in Table 1. The most commonly reported high-risk surgeries were general surgery (70.4%), cardiothoracic surgery (69.7%), and neurosurgery (62.5%), followed by orthopedic surgery (59.8%), gastrointestinal-hepatobiliary surgery (58.2%), obstetrics and gynecology (31.9%), and urology (27.2%) (Table 1).

The most frequently monitored parameters in high-risk surgeries were invasive blood pressure (96.3%), fluid balance (88.1%), and lactate levels (86.5%). Central venous pressure monitoring was used by 55.9% of respondents, while non-invasive blood pressure monitoring was reported by 53.1%. Among dynamic fluid management parameters, stroke volume variation (SVV), pulse pressure variation (PPV), and systolic pressure variation (SPV) were utilized by 32.1% of participants. CO monitoring was reported by only 12.6% of respondents (Table 2). Notably, 69% of respondents indicated that they never used CO monitoring in high-risk surgical patients.

Among those using CO monitoring, the PRAM-Mostcare monitor was the most commonly used device (62.2%), followed by PICCO (51.3%), Massimo LIDCO (28%), and transesophageal echocardiography (27%). The primary barrier to the use of CO monitoring was the high cost of the devices (68.8%), followed by the invasive nature of the available monitors (19.4%), difficulties in learning new monitoring techniques (18.9%), and increased workload (18.6%).

Table 3 summarizes the parameters used to assess fluid needs and evaluate volume replacement adequacy in high-risk surgical patients.

When all monitoring options were available, the preferred parameters for fluid deficit assessment and volume optimization are presented in Table 4.

More than half of the participants (59.3%, n = 331) reported never attending any training program on hemodynamic management or the use of advanced hemodynamic monitors. The most frequently reported challenge in applying advanced hemodynamic monitoring was difficulty in interpreting the monitored parameters (60.5%). Other significant challenges included difficulties in equipment use or placement (58.9%), concerns regarding device accuracy and reliability (58.2%), and the complexity of device interfaces (42.8%).

In cases of critical hemodynamic deterioration, the most frequently chosen intervention was the administration of cardiovascular drugs (74.5%). Other common responses included informing the surgical team (67.9%), applying additional monitoring methods (64.7%), adjusting the depth of anaesthesia (59.5%), and modifying the patient’s positioning (54.8%).

The general attitudes and behaviors of participants in extreme situations are summarized in Table 5. For awake high-risk surgical patients under regional anaesthesia, a significant proportion of respondents (15.1% and 37.1%) considered non-invasive blood pressure monitoring to be insufficient. Additionally, 41.1% of participants believed that SVV, PPV, and SPV parameters could be used to assess fluid deficits in awake patients. The vast majority (95.7%) agreed that hemodynamic monitors should be used for goal-directed therapy in pediatric patients. Furthermore, 60.1% of respondents believed that hemodynamic monitoring was valuable in guiding management even in patients with fluid overload (Table 5).

## Discussion

The results of this trial have revealed the limited usage of CO monitors among Turkish anaesthesiologists. The conventional parameters hold the majority of routine clinical practice, despite having the necessary knowledge regarding the indications of advanced hemodynamic monitorization. Such parameters like blood pressure, urine output, and lactate levels are still preferred over dynamic indices. Nevertheless, invasive blood pressure monitoring is the most frequently used method for close hemodynamic follow-up.

When this attitude is investigated within the world perspective, it can be seen more members of American Society of Anesthesiologists (35.4%) and European Society of Anaesthesiology and Intensive Care (34.9%) actively practice CO monitors than Turkish (12.6%) and Chinese (13.3%) communities, and this may be interpreted as technological advancements are more embraced in Western countries.^3, 4^ As CO guided goal-directed fluid therapy drops the incidence of intraoperative hypotension and perioperative complication rates, this massive gap in practice needs clarification.^8^ The long-term benefits of advanced hemodynamic monitorization is not observed intraoperatively, and one may argue that the physicians who are not included in the postoperative care may overlook the anticipated benefits of such monitorization.^9^ On the other hand, there is not a “sole” parameter that enhances the perioperative outcomes. Combination of several parameters is suggested to optimize CO, and in order to interpret those parameters correctly, a profound theoretical and physiological knowledge is required.^10^ Besides, the high costs and practical difficulties of these devices still stand as a handicap. Although hemodynamic principles are introduced during basic medical education, integrating this knowledge into clinical practice, interpreting relevant parameters, and applying them effectively can be challenging for many practitioners. This gap underscores the need for dedicated training programs aimed at enhancing the understanding of hemodynamics and the application of advanced monitoring techniques. In our study, a majority of participants reported that they had not received formal training in this area.

Abovementioned details might be accepted as an explanation for why advanced hemodynamic monitorization is not adapted to routine clinical practice, especially considering the busy environment of operating rooms. At this point, pulse contour analysis seem to be a solid option for hemodynamic follow-up since our results have validated broad use of invasive arterial blood pressure. SVV, SPV, and PPV are well-understood parameters by the majority of physicians (61%), yet very few use them routinely (32%) in order to distinguish volume responsiveness. Dynamic parameters are considered among the most reliable indicators for predicting fluid responsiveness, particularly in the context of goal-directed fluid therapy. However, their validity is highly dependent on specific physiological and mechanical ventilation conditions, such as regular rhythm, controlled mechanical ventilation, and absence of spontaneous breathing.^3, 5^ Although our findings indicate that participants generally understand the purpose of dynamic parameters, the notable rate of reported use under regional anaesthesia suggests a significant gap in knowledge regarding their limitations and the conditions required for accurate interpretation. This highlights the importance of targeted training programs that not only introduce these parameters but also emphasize the clinical scenarios in which they are appropriately applied.

Using advanced hemodynamic monitorization in goal-directed fluid therapy has been shown to be cost-effective for both hospital and communal economics.^11, 12^ Providing trainings and improving the basic knowledge regarding the principles of CO monitors would help administrators to gain a better insight on expected benefits which may consequently result in supplying these devices for physicians taking care of critical operations. Almost 75% of our participants were residents which explicitly indicates that advanced hemodynamics is not possibly embedded within the regular anaesthesiology training. Adapting such trainings into the curriculum might be one solid long-term solution with the aim of reducing perioperative morbidity and mortality. Anaesthesiologists should be able to interpret advanced parameters along with the mainstream ones. Another survey study from Italy has shown interesting results. Despite the high rates of CO monitor usage (41%), the physicians who do not use those have claimed they prefer to evaluate CO via central venous oxygen saturation (SvO₂) and mixed venous oxygen saturation (ScvO_2_).^6^ However, our results have revealed a very sparse use of SvO_2_ (19%) and ScvO_2_ (7%) among the Turkish anaesthesiologists which also supports the idea regarding the insufficient training on advanced hemodynamics.^13, 14, 15^

In high-risk patients undergoing awake surgery with regional anaesthesia, the use of CO monitoring may play a crucial role in guiding hemodynamic management. Studies have demonstrated that such advanced monitoring improves patient outcomes by optimizing fluid status, reducing the incidence of intraoperative hypotension, and ensuring more effective vasoactive drug administration.^16, 17^ Hemodynamic monitoring devices can be used for goal-directed therapy in critically ill pediatric patients. These devices provide real-time insights into cardiovascular parameters, such as CO, stroke volume, and systemic vascular resistance, which are essential for guiding fluid management, vasoactive drug administration, and other therapeutic interventions. In critically ill children, particularly those with complex or unstable conditions, advanced monitoring can help tailor individualized treatments, improving the likelihood of positive outcomes. Studies have demonstrated the potential benefits of using these devices in pediatric intensive care units, where early detection of hemodynamic changes and timely intervention are critical to improving survival rates and reducing morbidity.^18, 19^

Current study reflects a snapshot of routine clinical practice in Türkiye. Most survey trials that were referred above were performed at least one decade ago, and apparently, our clinical tendency is still away from the expected amendment, even though the reported outcomes are mainly from training hospitals (total 91%). Providing translational education via adapting basic sciences into routine training, and ensuring this teaching with hands-on practices would increase the general understanding of advanced hemodynamics. More importantly, as the existing literature mainly focus on the superiority of one parameter over another, simple diagrams and teaching materials that explain the hierarchy of such parameters are needed.^20^ The implementation of targeted educational interventions or specialized training programs in this area is expected to enhance both the conceptual understanding and clinical recognition of the critical role of hemodynamic monitoring.

### Study Limitations

This study has several limitations that should be considered when interpreting the findings. First, it is based on self-reported data, which may be subject to various biases, including overestimation or underestimation of actual practices and knowledge. Respondents may have provided socially desirable answers or may not have accurately recalled their routine clinical behaviors. Furthermore, the survey may not have captured all factors influencing hemodynamic monitoring practices, such as institutional policies, availability of resources, or individual clinician preferences. The cross-sectional nature of the study presents another limitation, as it offers a single time-point snapshot of current practices without capturing potential changes or trends over time. Additionally, while the survey achieved a relatively high response rate from anaesthesiologists working in university hospitals and training and research institutions, participation from other healthcare settings was limited. This may reflect a lower level of interest or engagement with the topic of hemodynamic monitoring in those populations, potentially leading to sampling bias. Given these limitations, the generalizability of our results to the entire population of anaesthesiologists in Türkiye is restricted. Nevertheless, the data provide valuable insights into prevailing attitudes and clinical tendencies regarding hemodynamic monitoring among a substantial segment of the anaesthesiology community.

## Conclusion

This study highlights the limited use of advanced hemodynamic monitoring techniques among anaesthesiologists in Türkiye, despite their recognized benefits in high-risk surgical patients. The predominant reliance on conventional parameters such as invasive blood pressure monitoring underscores the need for enhanced education and training in advanced hemodynamic monitoring. High costs, technical complexity, and lack of experience are significant barriers to the widespread adoption of these techniques. Addressing these challenges through targeted educational programs and practical training could improve the utilization of advanced hemodynamic monitoring, ultimately enhancing patient outcomes and reducing perioperative morbidity and mortality. The development of standardized protocols and nationwide algorithms may further support the integration of advanced hemodynamic monitoring into routine clinical practice.

## Ethics

**Ethics Committee Approval:** The study received approval from the Ankara Bilkent City Hospital, Scientific and Ethical Evaluation Board for Medical Research No. 2 (TABED) (approval no.: TABED 2-24-635, date: 13.11.2024).

**Informed Consent:** Informed consent was received from all participants.

## Supplementary Materials

Supplement Form 1 https://d2v96fxpocvxx.cloudfront.net/907e2968-841d-49d0-82d5-eec5fd9165af/content-images/66766dbc-4f73-4916-a9b2-6b501c4f155b.pdf

## Figures and Tables

**Table 1. Demographic and Descriptive Data of Participants:** 

**All participants (n = 713)**	**Respond rate number (%)**
**Age (years)**	
24-35	532 (74.6%)
36-45	84 (11.8%)
46-55	77 (10.8%)
>56	20 (2.8%)
**Gender**	
Female/Male	402/311 (56.3/43.7%)
**Work experience in anaesthesia (years)**	
0-5	486 (68.1%)
6-10	71 (10%)
11-20	99 (13.9%)
21-30	42 (5.9%)
>30	15 (2.1%)
**Number of operating rooms**	
<5	21 (2.9%)
5-10	45 (6.3%)
11-20	137 (19.2%)
21-30	198 (27.8%)
31-40	86 (12.1%)
>40	226 (31.7%)
**Type of the institution**	
State Hospital	45 (6.3%)
Training and Research Hospital	359 (50.4%)
University Hospital	293 (41.1%)
Private Hospital	16 (2.2%)
**Job title**	
Resident	503 (70.6%)
Specialist	122 (17.1%)
Faculty member	88 (12.3%)
**Frequency of anaesthesia management in high-risk surgeries**	
I do not directly manage anaesthesia	58 (8.1%)
1-5 times a week	364 (51.1%)
6-10 times a week	169 (23.7%)
>10 times a week	122 (17.1%)
**Timing of hemodynamic monitoring**	
Before anaesthesia induction	541 (75.9%)
After anaesthesia induction	456 (63.9%)
During surgery	191 (26.8%)
Postoperative	91 (12.7%)
**Distribution of high-risk surgeries**	
General Surgery	70.4%
Cardio-thoracic Surgery	69.7%
Neurosurgery	62.5%
Orthopedics and Traumatology	59.8%
Gastrointestinal and Hepatobiliary Surgery	58.2%
Obstetrics and Gynecology	31.9%
Urology	27.2%
Ear, Nose, Throat	14.3%
Others	3.9%

**Table 2. Routinely Used Hemodynamic Parameters in High-Risk Surgical Patients:** 

**Parameter (multiple choice available)**	**Respond rate number (%)**
Invasive blood pressure	96.3%
Fluid intake/uriner output	88.1%
Lactate	86.5%
Central venous pressure	55.9%
Non-invasive blood pressure	53.1%
Stroke volume variation, pulse pressure variation, systolic pressure variation	32.1%
P(a-v) CO_2_	21.7%
Near-infrared spectroscopy	21.1%
Central venous oxygen saturation	19.2%
Cardiac output	12.1%
Plethysmographic waveform variation	10.9%
Transesophageal echocardiography	10.5%
Mixed venous oxygen saturation (ScvO₂)	7.0%
Oxygen delivery (DO₂)	6.1%
Pulmonary capillary wedge pressure	1.6%
Esophageal Doppler (flow time corrected, FTc)	0.28%

**Table 3. Parameters Used to Determine Fluid Deficit and Fluid Adequacy:** 

**Fluid deficit (multiple choice available)**	**(%)**	**Fluid adequacy (multiple choice available)**	**(%)**
Urine output	95.0%	Increase in urine output	91.2%
Lactate	84.0%	Decrease in lactate	83.9%
Blood pressure	80.4%	Increase in blood pressure	82.3%
Clinical experience	78.3%	Decrease in heart rate	72.9%
Other blood gas parameters (base excess, pH)	70.8%	Decrease in SVV, PPV, SPV	50.8%
SVV, PPV, SPV	60.9%	Other blood gas parameters (base excess, pH)	49.5%
CVP	53.0%	Increase in cardiac output	30.2%
Passive leg raise and fluid challenge	51.9%	Decrease in SVR	20.7%
Cardiac output	25.6%	Decrease in PVI	17.8%
Ultrasound/echocardiography	22.1%	Increase in ScvO₂	12.2%
Pleth variable index	15.4%	Decrease in P(a-v) CO₂	10.8%
P(a-v) CO_2_	12.2%	Increase in SvO_2_	10%
ScvO_2_	9.3%	-	-
SvO_2_	6.8%	-	-

Total number of respondents: 628

CVP, central venous pressure; SVV, stroke volume variation; PPV, pulse pressure variation; SPV, systolic pressure variation; SvO₂, central venous oxygen saturation; ScvO₂, mixed venous oxygen saturation; SVR, systemic vascular resistance

**Table 4. Preferred Parameters for Fluid Deficit and Optimization in the Presence of All Available Resources:** 

**Parameter (multiple choice available)**	**%**
Urine output	84.0%
Clinical experience	78.8%
SVV, PPV, SPV	76.3%
Cardiac output	72.9%
Blood pressure	71.8%
Transesophageal echocardiography	52.8%
Central venous pressure	52.8%
Plethysmographic waveform variation	37.1%
Near-infrared spectroscopy	35.8%
Central venous oxygen saturation	32.6%
Mixed venous oxygen saturation	31.0%
Pulmonary capillary wedge pressure	22.2%

Total number of respondents: 558

SVV, stroke volume variation; PPV, pulse pressure variation; SPV, systolic pressure variation

**Table 5. General Attitudes and Behaviors of Participants:** 

**Statement**	**Strongly disagree**	**Disagree**	**Somewhat agree**	**Agree**	**Strongly agree**	**Total**
Non-invasive arterial blood pressure monitoring is sufficient for hemodynamic monitoring in high-risk surgical patients under regional anaesthesia.	15.1% 84	37.1% 206	37.9% 210	8.3% 46	1.6% 9	555
Stroke volume monitoring with invasive arterial blood pressure in high-risk patients under regional anaesthesia guides fluid and vasoactive drug therapy.	1.9% 11	5.1% 29	28.1% 156	54.9% 306	10% 55	557
SVV, PPV, and SPV can be used to assess fluid responsiveness in awake patients.	9.7% 54	13.3% 74	30.4% 168	41.2% 228	5.4% 30	554
Hemodynamic monitoring devices can be used for goal-directed therapy in critically ill pediatric patients.	0.4% 2	3.9% 22	25.6% 142	53.5% 297	16.6% 92	555
Hemodynamic monitors are useful in deciding erythrocyte suspension replacement in high-risk patients.	1.1% 6	5.7% 32	23.5% 131	55.7% 311	14% 78	558
Advanced hemodynamic monitoring also provides an advantage in the management of patients with fluid overload.	0.4% 2	1.4% 8	14.4% 80	60.1% 335	23.7% 132	557

SVV, stroke volume variation; PPV, pulse pressure variation; SPV, systolic pressure variation
